# Effect of a physical activity and healthy eating lifestyle intervention in pregnancy on fetal growth trajectories: The DALI randomised controlled trial

**DOI:** 10.1111/ijpo.13199

**Published:** 2025-01-19

**Authors:** Anna M. Dieberger, Mireille N. M. van Poppel, Gernot Desoye, David Simmons, Jürgen Harreiter, Roland Devlieger, Carmen Medina, Deborah A. Lawlor, Ahmed Elhakeem, the DALI core investigator group, Gernot Desoye, David Simmons, Rosa Corcoy, Juan M. Adelantado Perez, Alexandra Kautzky‐Willer, Jürgen Harreiter, Elizabeth Mathiesen, Dorte M. Jensen, Lise Lotte T. Andersen, Fidelma Dunne, Annunziata Lapolla, Maria G. Dalfra, Alessandra Bertolotto, Mireille N. M. van Poppel, Judith G. M. Jelsma, Sander Galjaard, Ewa Wender‐Ozegowska, Agnieszka Zawiejska, David Hill, Roland Devlieger, Frank J. Snoek

**Affiliations:** ^1^ Department of Obstetrics and Gynaecology Medical University of Graz Graz Austria; ^2^ Department of Human Movement Science, Sport and Health University of Graz Graz Austria; ^3^ Macarthur Clinical School Western Sydney University Campbelltown Australia; ^4^ Division of Endocrinology and Metabolism, Department of Medicine III Medical University of Vienna Vienna Austria; ^5^ Department of Medicine Landesklinikum Scheibbs Scheibbs Austria; ^6^ Department of Development and Regeneration, Faculty of Medicine Katholieke Universiteit Leuven Leuven Belgium; ^7^ Department of Obstetrics and Gynaecology University Hospitals Leuven Leuven Belgium; ^8^ Department of Obstetrics and Gynaecology Hospital de la Santa Creu I Sant Pau Barcelona Spain; ^9^ MRC Integrative Epidemiology Unit at the University of Bristol Bristol UK; ^10^ Population Health Sciences, Bristol Medical School University of Bristol Bristol UK

**Keywords:** foetal growth, intervention, lifestyle, ultrasound

## Abstract

**Background:**

Obesity during pregnancy is related to fetal overgrowth. Effective interventions that can mitigate this risk are needed.

**Objectives:**

This study aimed to investigate the effect of a lifestyle intervention for pregnant women with obesity on fetal growth trajectories.

**Methods:**

In the DALI trial, pregnant women with a body mass index ≥29.0 kg/m^2^ and without gestational diabetes at baseline were randomized to counselling on physical activity (PA), healthy eating (HE) or a combination (PA + HE), or to usual care (UC). Fetal growth trajectories were modelled based on a combination of estimated fetal weight (EFW) from repeated ultrasound scans and weight measured at birth. Differences in fetal growth trajectories between groups were assessed.

**Results:**

Three hundred eighty‐four women were included. Those in the PA + HE intervention had slower EFW gain from 32 weeks onwards, with differences (PA + HE vs. UC) at 32, 36 and 40 weeks of −54.1 g (−146.7 to 38.9 g), −84.9 g (−194.0 to 24.7 g), and −99.8 g (−227.1 to 28.1 g), respectively. Effects appeared stronger in males, with a difference at 40 weeks of −185.8 g (−362.5 g to −9.2 g) versus −23.4 g (−190.4 g to 143.5 g) in females.

**Conclusions:**

A lifestyle intervention for pregnant women with obesity resulted in attenuated fetal growth, which only reached significance in male offspring. Future larger trials are needed to confirm these findings and elucidate underlying pathways.

## 1 INTRODUCTION

The prevalence of overweight and obesity among women of reproductive age is increasing.^1^, ^2^ Maternal obesity is related to adverse health outcomes for mothers and offspring, such as maternal glucose intolerance and related fetal overgrowth,^3^ and consequently higher birthweight and large for gestational age (LGA) offspring.^4^, ^5^

Interventions to reduce the adverse perinatal effects of overweight and obesity in pregnant women have been developed and tested,^6^ including the vitamin D And Lifestyle Intervention for gestational diabetes mellitus prevention (DALI) randomised controlled trial (RCT). It specifically targeted pregnant women with overweight and obesity with the primary aim to reduce gestational weight gain (GWG) and fasting glucose and improve insulin sensitivity in late pregnancy through lifestyle modifications.^7^ The DALI lifestyle trial randomized women into four different groups receiving counselling either on physical activity (PA) or healthy eating (HE), counselling for both (PA + HE) or receiving usual care (UC). Previous analyses showed that, while all three intervention groups improved lifestyle behaviour, no effects on fasting glucose or insulin sensitivity were achieved.^8^ The PA + HE group, however, showed a reduction in gestational weight gain^8^ and reduced adiposity in neonates, the latter effect mediated by a reduction in maternal sedentary time.^9^

Differences in neonatal weight and body composition at birth are the result of differences in fetal growth. The foetus gains most weight during the second half of gestation,^10^ with body fat accumulation starting at around 23 weeks of gestation and rising steeply until birth.^11^ Studies have shown how unfavourable intrauterine environments affect fetal growth. It is well known that offspring of mothers with obesity or hyperglycaemia have higher estimated fetal weight (EFW) from 20 to 30 weeks of gestation onward.^12^, ^13^, ^14^, ^15^ However, less recognized is the biphasic pattern of growth deviation in fetuses of women with diabetes or obesity compared with those of lean women,^16^ with a growth reduction in early pregnancy and excessive foetal growth in late pregnancy. This makes assessing the effect of interventions on fetal growth at different gestational ages relevant. Furthermore, it has been reported that intervention effects on maternal metabolism are dependent on the timing of initiating the intervention.^17^ Therefore, it is tempting to hypothesize that this timing also determines whether an intervention is effective in reducing excessive fetal growth or adiposity in women with obesity or GDM. The TOBOGM (Treatment Of BOoking GdM) RCT has recently shown that treating early GDM (diagnosed before 20 weeks of gestation) leads to a reduction in neonatal fat mass at birth,^18^ but it did not assess when this treatment effect on fetal growth starts.

Because the combined PA + HE intervention was initiated at around 16 weeks of gestation and effectively reduced neonatal adiposity, the DALI study is well suitable to assess when in pregnancy, a change in fetal growth trajectory can be found. The aim of this study was to test the hypothesis that a pregnancy lifestyle intervention for women with overweight or obesity affects fetal growth trajectories across pregnancy.

## 2 METHODS

### 2.1 Design and participants

The DALI study was designed as a multicentre RCT with a 2 × (2 × 2) factorial design consisting of a vitamin D and lifestyle trial arm.^7^ This is a secondary analysis of the lifestyle trial only, and none of the women included received vitamin D supplementation. The trial was conducted in Austria, Belgium, Denmark (Copenhagen, Odense), Ireland, Italy (Pisa, Padua), the Netherlands, Poland, Spain and the United Kingdom between 2012 and 2015. Women with a prepregnancy body mass index (BMI) of ≥29 kg/m^2^, a singleton pregnancy prior to 20 weeks of gestation and aged ≥18 years were asked to participate. Those with preexisting diabetes mellitus and other chronic medical conditions were excluded. Women with early gestational GDM on oral glucose tolerance test (OGTT) according to IADPSG (International Association of the Diabetes and Pregnancy Study Groups) criteria^19^ were excluded before randomization. Only women with fetal ultrasound data available were included in this secondary analysis.

The DALI RCT was prospectively registered as ISRCTN70595832. All relevant local ethics committees approved of the study, and written informed consent was acquired from all participants. This study followed the CONSORT reporting guidelines.

### 2.2 Interventions and control

In the lifestyle trial, women were randomised by a computerised random number generator which was pre‐stratified by study site. Participants were randomised equally into three intervention groups, consisting of PA counselling, HE counselling, or combined counselling for both (PA + HE), or a fourth control group receiving UC. While blinding of study participants was not possible, the measurement team was blinded to the randomisation.

After randomisation, all participants of the intervention groups received five face‐to‐face, individual coaching sessions with a single lifestyle coach, lasting 30–45 min. The sessions were alternated with up to four additional telephone booster calls. The first session was planned before 20 weeks, and the majority of the coaching intervention, that is four of the five face‐to‐face sessions, needed to take place before the first follow‐up measurement at 24–28 weeks of gestation. The intervention was built on principles of motivational interviewing (MI) and built on five main messages for the PA intervention, including advice to reduce sedentary time and be more active, increase number of steps and build up strength and seven main messages for the HE intervention, including advice on reducing carbohydrate and fat intake and increasing vegetable, fibre and protein intake. The PA + HE group received the content of both the PA as well as the HE intervention. The UC group received no intervention but usual pregnancy care.

Prior to the study, the lifestyle coaches delivering the intervention received a 2‐day central training course in Cambridge (UK), which was provided by experienced MI trainers.

### 2.3 Measurements

#### 2.3.1 General variables

Maternal height was measured at baseline (<20 weeks) using a stadiometer (SECA 206; SECA, Birmingham, UK). Prepregnancy weight was determined by questionnaire. Weight during pregnancy was measured at baseline, 24–28 weeks and 35–37 weeks with calibrated electronic scales (SECA 888 and 877). BMI was calculated as weight (kg) divided by height squared (in square meter). GWG was defined as the difference in objectively measured weight between baseline and 35–37 weeks.

Characteristics such as parity (nulliparity vs. multiparity), education (university level vs. lower), ethnicity (European vs. non‐European descent), domestic circumstances (living with partner vs. living alone), prenatal smoking and alcohol intake (yes vs. no), paternal height, mode of delivery (caesarean section vs. vaginal delivery), hypertensive disorders of pregnancy including pregnancy‐induced hypertension and pre‐eclampsia (yes vs. no) and gestational age at birth were collected by questionnaire or extracted from medical files.

At 24–28 weeks, a repeat OGTT was performed to diagnose GDM. Those who showed normal glucose tolerance were asked to repeat the test at 35–37 weeks.

#### 2.3.2 Fetal growth measurements

Ultrasound scans were performed repeatedly through pregnancy, with at least three scans (during the first trimester, at 24–28 weeks and 35–37 weeks) required as part of the study protocol. One early ultrasound (<14 weeks of gestation) was mandatory to define crown rump length to acquire an accurate estimated date of gestation. Depending on the study site, scans were either part of standard care or performed specifically for the study. Information on further ultrasound scans performed at additional time points as part of usual prenatal care was also collected. Ultrasound scans were performed as defined by the International Society of Ultrasound in Obstetrics and Gynaecology guidelines.^20^

At each ultrasound scan, fetal biometry, including biparietal head diameter, head circumference (HC), abdominal circumference (AC) and femur length (FL), was measured to the nearest millimetre. EFW (in grams) was calculated according to the Hadlock formula^21^: log_10_ EFW = 1.326–0.00326 * AC * FL + 0.0107 * HC + 0.0438 * AC + 0.158 * FL.

Birthweight (in grams) was measured at birth;  within 48 h, neonatal AC and HC (in millimetre) were measured and combined with foetal anthropometric data to allow for the estimation of growth trajectories. Small‐for‐gestational age (SGA) and LGA were defined as <10th and >90th birthweight percentiles, respectively.

### 2.4 Statistical analysis

Power calculations were conducted for the original RCT,^7^ based on the primary outcomes of gestational weight gain, fasting glucose and insulin sensitivity in late pregnancy. Data were analysed according to the intention‐to‐treat principle. Participants who did not finish the trial, dropped out or were lost‐to‐follow‐up were included in all analyses if at least one ultrasound measurement was available. Statistical analyses for this paper were performed from September 2021 until February 2022.

#### 2.4.1 Descriptive statistics

Maternal and neonatal characteristics are presented by randomisation group. In addition, included participants were compared with those excluded due to missing ultrasound data. Characteristics were also compared between participants with different numbers of repeat ultrasound measures.

#### 2.4.2 Longitudinal analyses

Fetal growth trajectories were derived using natural cubic spline multilevel models^22^ with two levels, clustering measurement occasions (Level 1) within individuals (Level 2). Through this approach, within‐person correlations in repeat measures are accounted for. All those with at least one ultrasound measurement were included under the missing at random assumption.

As fetal growth is not linear, natural cubic splines with two knots were applied to find best‐fitting trajectories conditioned on gestational age for each growth parameter. A random intercept and random slope were added at Level 2 to allow for individual growth trajectories. Study centre was added as covariate. To account for differences in measurement error between ultrasound and birth measurements, level 1 residuals were allowed to vary over time.

Differences in growth trajectories by intervention group were analysed by adding the randomisation group variables and their interactions with gestational age to the models as independent variables. Mean fetal size of each growth parameter was estimated at 4‐week intervals between 16 and 40 weeks of gestation for each randomisation group, and differences between mean fetal size in the intervention groups and the UC group were calculated.

To test for effect modification by fetal sex, the variable and interaction terms with the gestational age and randomization group variables were added.

#### 2.4.3 Sensitivity analyses

As in the main RCT, the main analyses here compared each of the three intervention arms (PA + HE, PA alone, HE alone) individually to UC. In a sensitivity analysis, we combined participants from any of the three intervention groups and compared them to UC, to increase power. Two additional sensitivity analyses were performed, firstly adjusting for the number of growth measurements and secondly adjusting for the type of measurement (fetal ultrasound vs. neonatal).

Modelling was performed using the *nlme* package (version 3.1–153)^23^ in *R* (version 4.0.4).^24^

## 3 RESULTS

### 3.1 Participant characteristics

**FIGURE 1:**
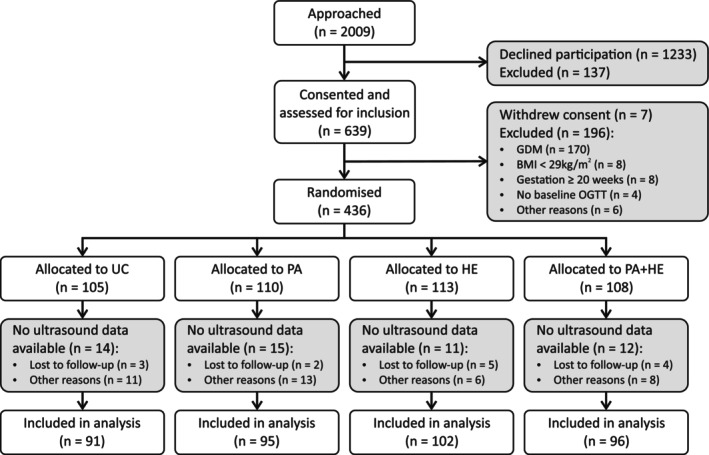
CONSORT flow diagram. BMI, body mass index; GDM, gestational diabetes mellitus; HE, healthy eating intervention; OGTT, oral glucose tolerance test; PA, physical activity intervention; UC, usual care.

Of the original 436 randomised participants of the lifestyle trial, 384 had at least one ultrasound measurement available (PA + HE: *n* = 96, PA: *n* = 95, HE: *n* = 102, UC: *n* = 91) and were included in this analysis (Figure 1). Maternal and neonatal characteristics were similar in the randomisation groups (Table 1), except for gestational weight gain, which, concordant with the main findings of the trial,^8^ was significantly reduced in the PA + HE group. We also found a lower birthweight (3445 g in HE and 3470 g in PA + HE vs. 3541 g in UC) and a lower number of LGA neonates in the combined PA + HE group (8% vs. 15% in UC), although differences were not statistically significant. The number of participants without scans was comparable across randomisation groups (Table S1). Besides excluded participants being taller, maternal characteristics of included and excluded participants were similar.

**TABLE 1:** Maternal and neonatal characteristics by randomization group (No. = 384).

Variable	No.	UC (No. = 91)	No.	PA (No. = 95)	No.	HE (No. = 102)	No.	PA + HE (No. = 96)
Maternal characteristics								
Age, mean (SD), years	91	31.8 (5.5)	95	31.5 (5.1)	102	32.4 (5.5)	96	32.2 (5.1)
Multiparous, No. (%)	91	43 (47)	95	44 (46)	102	59 (58)	96	48 (50)
Higher education, No. (%)	91	49 (54)	95	50 (53)	102	57 (56)	96	54 (56)
European descent, No. (%)	91	81 (89)	95	79 (83)	102	85 (83)	96	83 (86)
Living with partner, No. (%)	91	87 (96)	95	89 (94)	102	99 (97)	96	88 (92)
Smoking, No. (%)	91	16 (18)	94	16 (17)	102	20 (20)	96	11 (11)
Alcohol consumption, No. (%)	91	6 (7)	94	3 (3)	102	8 (8)	96	5 (5)
Height, mean (SD), cm	91	165.4 (6.5)	95	165.5 (7.3)	102	164.7 (6.5)	96	165.7 (6.8)
Prepregnancy BMI, median (IQR), kg/m^2^	91	33.0 (30.8, 35.6)	95	32.5 (30.4, 35.9)	102	32.6 (30.8, 35.3)	96	33.1 (30.7, 36.0)
GWG at 35–37 weeks, mean (SD), kg	81	8.5 (4.8)	79	8.3 (5.0)	84	7.6 (4.6)	83	6.2 (3.9)
GDM at 24–28 or 35–37 weeks, No. (%)	84	33 (39)	79	27 (34)	84	37 (44)	81	27 (33)
Hypertensive disorders of pregnancy, No. (%)	85	11 (13)	84	12 (14)	96	14 (15)	81	11 (14)
Available ultrasound measurements, No. (%)	91		95		102		96	
1–3		73 (80)		70 (74)		80 (78)		70 (73)
4–6		11 (12)		15 (16)		15 (15)		19 (20)
7–10		7 (8)		10 (11)		7 (7)		7 (7)
Neonatal characteristics								
Female sex, No. (%)	88	47 (53)	87	43 (49)	99	45 (45)	89	46 (52)
Gestational age at birth, median (IQR), weeks	86	40.0 (38.7, 41.1)	87	39.6 (38.9, 40.6)	98	39.6 (38.6, 40.8)	88	39.8 (38.9, 40.7)
Preterm birth (<37 gestational weeks), No. (%)	86	5 (6)	84	4 (5)	96	8 (8)	89	2 (2)
Caesarean section, No. (%)	87	30 (34)	86	26 (30)	98	35 (36)	89	26 (29)
Birthweight, mean (SD), g	88	3541 (520)	88	3506 (478)	99	3445 (653)	89	3470 (504)
Small for gestational age, No. (%)	86	5 (6)	84	3 (4)	98	10 (10)	85	7 (8)
Large for gestational age, No. (%)	86	13 (15)	85	12 (14)	98	15 (15)	85	7 (8)
Abdominal circumference at birth, mean (SD), mm	74	334 (26)	74	331 (28)	76	334 (32)	69	332 (25)
Head circumference at birth, mean (SD), mm	85	348 (17)	82	351 (16)	91	346 (20)	85	348 (15)

*Note*: Randomisation groups: HE, healthy eating intervention; PA, physical activity intervention; PA + HE, physical activity and healthy eating intervention; UC, usual care.

Abbreviations: BMI, body mass index; GDM, gestational diabetes mellitus; GWG, gestational weight gain; No., number of participants.

The median (interquartile range) number of anthropometric measurements per fetus was 4 (4–4), and mean gestational age at the first fetal scan was 14.9 (SD 4.9) weeks. The number of scans per gestational period can be found in Table S2. When comparing participants with different numbers of ultrasound scans (Table S3), those receiving the most scans (7–10 scans) had higher rates of pregnancy complications such as hypertensive disorders (29% compared with 15% and 12% in those with 4–6 and 1–3 scans) and GDM (50% compared with 46% and 34% in those with 4–6 and 1–3 scans) and more often birthed LGA offspring (29% in those with 7–10 scans compared with 14% and 11% with 3–6 and 1–3 scans). Women receiving more ultrasounds throughout pregnancy on average had lower education, were more often of non‐European descent and were more often living without a partner.

### 3.2 Growth over time

EFW throughout pregnancy increases in a curvilinear way, showing incremental growth in the second half of pregnancy (Figure S1). HC, AC and FL growth trajectories appear almost linear throughout pregnancy, with a decline in growth towards the end of pregnancy.

**FIGURE 2:**
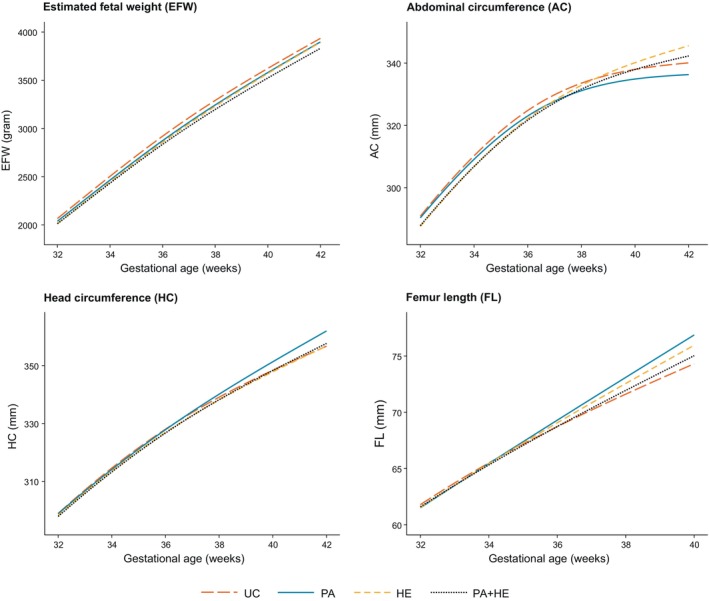
Fetal growth trajectories from 32 weeks of gestation onwards per randomisation group. Predicted mean growth trajectories of EFW (estimated fetal weight; in grams), HC (head circumference; in millimetre), AC (abdominal circumference; in millimetre) and FL (femur length; in millimetre) stratified by randomisation group: UC (usual care), PA (physical activity intervention), HE (healthy eating intervention) and PA + HE (physical activity and healthy eating intervention) from 32 weeks of gestation onwards. Growth trajectories were estimated using multilevel natural cubic spline models with 2 knots containing an interaction term between gestational age at measurement (continuous; weeks) and randomisation group (categorical; UC, PA, HE, PA + HE). Study site (categorical; Austria, Belgium, Denmark [Copenhagen, Odense], Ireland, Italy [Pisa, Padua], Netherlands, Poland, Spain, United Kingdom) was added to all models as covariate with Spain set as reference category.

**FIGURE 3:**
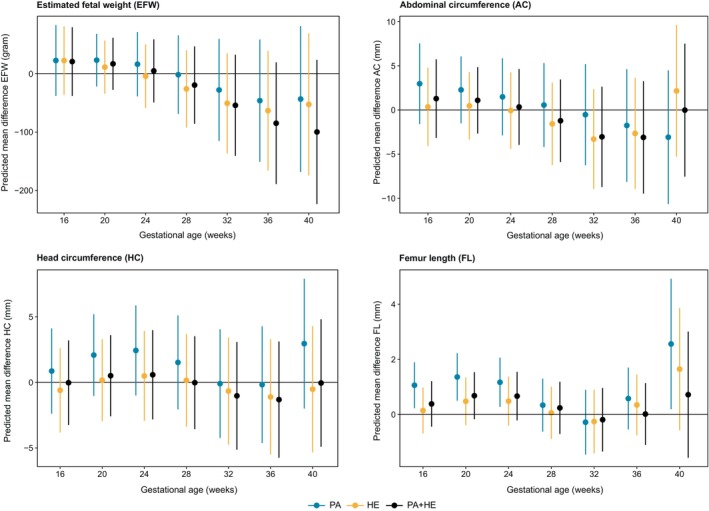
Intervention effect on fetal size across gestation. Predicted mean differences in mean EFW (estimated fetal weight; in grams), HC (head circumference; in millimetre), AC (abdominal circumference; in millimetre) and FL (femur length; in millimetre) comparing the interventions PA (physical activity), HE (healthy eating) and PA + HE (physical activity and healthy eating) to UC (usual care; reference group) across gestation. Mean differences were estimated using multilevel natural cubic spline models with 2 knots containing an interaction term between gestational age at measurement (continuous; weeks) and randomisation group (categorical; UC, PA, HE, PA + HE). Study site (categorical; Austria, Belgium, Denmark [Copenhagen, Odense], Ireland, Italy [Pisa, Padua], Netherlands, Poland, Spain, United Kingdom) was added to all models as covariate.

The PA + HE intervention resulted in nonsignificant, slower growth of EFW (Figures 2 and 3; Table 2; Figure S2; Table S4) from 32 weeks onwards compared with UC, with differences increasing with gestation (−54.1 g, 95% CI: −146.7 to 38.9 g at 32 weeks and −84.9 g, −194.0 to 24.7 g at 36 weeks). At 40 weeks, the predicted mean difference in EFW was −99.8 g (−227.1 to 28.1 g). Differences between PA or HE alone compared with UC were smaller than those between the combined intervention and UC (Figures 2 and 3; Table 2; Figure S2; Table S4). Predicted mean differences at 40 weeks for PA versus UC and HE versus UC were −43.4 g (−172.8 to 85.9 g) and −52.6 g (−178.5 to 73.6 g).

**TABLE 2:** Predicted mean differences in fetal size across gestation comparing intervention groups to usual care.

	Predicted mean difference compared with UC (95% CI)		
GA	PA	HE	PA + HE
EFW, g (No. = 377; *n* = 1337)			
16 weeks	22.7 (−48.3; 93.6)	22.5 (−47.6; 92.2)	20.8 (−48.0; 89.9)
20 weeks	23.2 (−34.5; 81.1)	11.3 (−46.7; 69.4)	17.0 (−39.3; 73.8)
24 weeks	16.3 (−49.3; 82.0)	−4.2 (−68.8; 60.7)	4.8 (−58.6; 69.0)
28 weeks	−1.6 (−77.6; 74.4)	−26.0 (−100.2; 48.6)	−19.5 (−93.3; 55.1)
32 weeks	−27.9 (−121.9; 66.1)	−50.6 (−142.8; 41.6)	−54.1 (−146.7; 38.9)
36 weeks	−46.2 (−156.4; 64.1)	−63.3 (−171.4; 44.7)	−84.9 (−194.0; 24.7)
40 weeks	−43.4 (−172.8; 85.9)	−52.6 (−178.5; 73.6)	−99.8 (−227.1; 28.1)
AC, mm (No. = 372; *n* = 1289)			
16 weeks	3.0 (−1.6; 7.6)	0.4 (−4.1; 4.8)	1.3 (−3.2; 5.7)
20 weeks	2.3 (−1.5; 6.1)	0.5 (−3.3; 4.3)	1.1 (−2.7; 4.9)
24 weeks	1.5 (−2.9; 5.9)	−0.1 (−4.4; 4.3)	0.3 (−4.0; 4.6)
28 weeks	0.6 (−4.2; 5.3)	−1.6 (−6.2; 3.1)	−1.2 (−5.9; 3.5)
32 weeks	−0.5 (−6.3; 5.2)	−3.3 (−9.0; 2.4)	−3.0 (−8.7; 2.6)
36 weeks	−1.8 (−8.1; 4.6)	−2.7 (−9.0; 3.6)	−3.1 (−9.5; 3.3)
40 weeks	−3.1 (−10.7; 4.5)	2.2 (−5.3; 9.6)	0.0 (−7.6; 7.5)
HC, mm (No. = 379; *n* = 1365)			
16 weeks	0.9 (−2.4; 4.1)	−0.6 (−3.8; 2.6)	0.0 (−3.2; 3.2)
20 weeks	2.1 (−1.0; 5.2)	0.2 (−3.0; 3.3)	0.5 (−2.6; 3.6)
24 weeks	2.4 (−1.0; 5.9)	0.5 (−2.9; 3.9)	0.6 (−2.8; 4.0)
28 weeks	1.5 (−2.1; 5.1)	0.2 (−3.4; 3.7)	0.0 (−3.6; 3.5)
32 weeks	−0.1 (−4.2; 4.1)	−0.7 (−4.7; 3.4)	−1.0 (−5.1; 3.1)
36 weeks	−0.2 (−4.6; 4.3)	−1.1 (−5.5; 3.3)	−1.3 (−5.7; 3.1)
40 weeks	3.0 (−2.0; 7.9)	−0.5 (−5.3; 4.3)	0.0 (−4.9; 4.8)
FL, mm (No. = 376; *n* = 1043)			
16 weeks	1.1 (0.2; 1.9)	0.1 (−0.7; 1.0)	0.4 (−0.4; 1.2)
20 weeks	1.4 (0.5; 2.2)	0.5 (−0.4; 1.3)	0.7 (−0.2; 1.5)
24 weeks	1.2 (0.3; 2.1)	0.5 (−0.4; 1.4)	0.7 (−0.2; 1.5)
28 weeks	0.3 (−0.6; 1.3)	0.1 (−0.9; 1.0)	0.2 (−0.7; 1.2)
32 weeks	−0.3 (−1.5; 0.9)	−0.3 (−1.4; 0.9)	−0.2 (−1.3; 1.0)
36 weeks	0.6 (−0.5; 1.7)	0.3 (−0.8; 1.4)	0.0 (−1.1; 1.1)
40 weeks	2.6 (0.2; 4.9)	1.6 (−0.6; 3.9)	0.7 (−1.6; 3.0)

*Note*: Predicted mean differences in EFW (estimated fetal weight; in grams), HC (head circumference; in millimetre), AC (abdominal circumference; in millimetre) and FL (femur length; in millimetre) comparing the interventions PA (physical activity), HE (healthy eating) and PA + HE (physical activity and healthy eating) to UC (usual care; reference group) across gestation. Mean differences were estimated using multilevel natural cubic spline models with 2 knots containing an interaction term between gestational age at measurement (continuous; weeks) and randomisation group (categorical; UC, PA, HE, PA + HE). Study site (categorical; Austria, Belgium, Denmark [Copenhagen, Odense], Ireland, Italy [Pisa, Padua], Netherlands, Poland, Spain, United Kingdom) was added to all models as covariate. No., number of participants; *n*, number of observations. See Figure 3 for graphical presentation of results.

In all intervention groups, fetal growth trajectories of AC and HC were similar to the UC group (Figures 2 and 3; Table 2; Figure S2; Table S4).

From early pregnancy until 24 weeks as well as at 40 weeks of gestation, FL growth was increased in foetuses in the PA group compared with the UC group. However, as no neonatal measurements were available for FL, predictions in late pregnancy were based on a very limited number of measurements. Fetal FL in the HE and PA + HE groups were not significantly different than those in the UC group.

### 3.3 Differences by fetal sex

When stratifying predictions by fetal sex, the effect of PA + HE on EFW was driven mainly by male offspring, who showed decreased EFW growth from 32 weeks' gestation onwards compared with UC (Figure S3; Tables S5 and S6; mean difference −185.8 g [−362.5 to −9.2 g] in males and −23.4 g [−190.4 to 143.5 g] in females at 40 weeks). Similar sex‐specific effects were found for AC growth trajectories. No clear differences in HC or FL growth trajectories by intervention group were found in either males or females.

### 3.4 Sensitivity analyses

When comparing the three intervention groups combined to the control group (Figure S4; Table S7), growth of EFW was attenuated in the intervention groups from 28 weeks' gestation onwards and decreased further towards the end of pregnancy; however, confidence intervals were wide and included the null at all ages. For example, at 28 weeks, the mean difference was −15.8 g (−74.1 to 42.5 g) and increased to −65.4 g (−168.9 to 38.1 g) at 40 weeks. No clear differences were found comparing growth of AC and HC between both groups. Like in the main analyses, FL growth was increased in the intervention group compared with UC throughout most of pregnancy.

Sensitivity analyses with adjustment for number of measurements per fetus (Table S8) and type of measurement (ultrasound scan vs. neonatal anthropometry, Table S9) did not change results.

## 4 DISCUSSION

The findings of our RCT suggest that a lifestyle intervention in pregnant women with overweight or obesity may attenuate growth in fetal weight when compared with UC, but estimates were imprecise with wide confidence intervals that included the null. The effect was mainly driven by male offspring.

We found longer FL in the PA group compared with those not receiving the intervention, but as differences were detected as early as 16 weeks of gestation and thus prior to the start of the intervention, this is likely a coincidental finding.

Similar to our findings, in the LIMIT trial, an intervention consisting of dietary and lifestyle advice did not result in significant differences in EFW, HC or FL.^25^ However, greater mid‐thigh fat mass and a slower rate of subscapular adipose tissue deposition among fetuses in the intervention group were reported, which the authors interpret as indicating a more favourable distribution of body fat. Both the LIMIT and UPBEAT trial reported on birth size in offspring of women with overweight or obesity.^26^, ^27^ While they found no difference in LGA^26^, ^27^ or mean birthweight,^26^ the LMIIT trial found reduced macrosomia (birthweight ≥4000 g) in the intervention arm.^27^, ^28^ An RCT investigating the effect of a low glycaemic dietary intervention in women with a previous macrosomic offspring found no differences in offspring outcomes (mean birthweight, birthweight ≥4000 g, ultrasonic EFW, AC, or abdominal wall thickness at 34 weeks of gestation).^29^ Size‐ and weight‐related outcomes might not be very sensitive in detecting changes in body composition due to maternal lifestyle changes. Therefore, including measures of offspring adiposity in the evaluation of lifestyle interventions in pregnancy is warranted.

One of the main messages of the HE intervention was advice to reduce carbohydrate intake and both the HE and PA + HE group managed to reduce their carbohydrate and sugared drinks intake.^8^ A South African observational study found increased fetal growth in mothers with a dietary pattern high in sugar.^30^ Interestingly, similar to our RCT, when stratifying by fetal sex, associations between diet and fetal growth were only found in males in that study. That we saw smaller differences in the HE group compared with UC might be due to a synergistic or additive effect of reducing sedentary time. We previously reported mediation by reduced sedentary time on neonatal adiposity,^9^ which could indicate that this behaviour is relevant for fetal growth. Another key message for all intervention groups was to strive for a maximum GWG of 5 kg, but only the PA + HE group achieved a reduced GWG compared with UC (−2.02 kg, −3.58 to −0.46 kg). This might have contributed to the attenuated fetal growth in this group, although the two largest studies to date using the MOCO/Lifecycle collaboration (*N* = 162 129 and 196 670) show that maternal prepregnancy BMI has a stronger association than GWG with birth size, other pregnancy and neonatal outcomes and future offspring BMI.^31^, ^32^

Whether the difference in fetal growth trajectories found here is clinically relevant, is difficult to establish, because this will depend on long‐term outcomes. So far, lifestyle interventions in pregnancy for women with overweight or obesity have not resulted in reduced offspring childhood obesity risk.^33^ In the DALI PA + HE intervention group, birthweight was reduced by ~100g,^8^ mostly accounted for by reduced neonatal fat mass (−63 g vs. UC).^9^ In a small‐scale study^34^ and the large HAPO follow‐up,^35^ adiposity at birth was associated with adiposity and risk of obesity in childhood. Therefore, we are currently hypothesizing that the reduction of fetal growth and neonatal fat found in the DALI study may associate with reduced risk of obesity in childhood. The reduction in foetal growth in the PA + HE intervention group was not related to adverse pregnancy and birth outcomes, because we did not see differences in SGA, preterm birth, neonatal hypoglycaemia or NICU admission between intervention groups.

The association between maternal glucose metabolism and fetal growth has long been established. Initiating interventions before 15 weeks of gestation has been shown to be essential for an effect on maternal glucose metabolism^17^, ^36^ and might explain why the DALI lifestyle interventions failed to significantly influence fetal growth.

Excess fetal growth has been found to be incremental in relation to maternal fasting glucose.^13^, ^16^, ^37^, ^38^, ^39^ Maternal hyperglycaemia is related to foetal hyperinsulinism.^40^ Foetal insulin is a growth factor and is estimated to determine about 50% of birthweight.^41^ There is evidence that male neonates are more insulin sensitive than females.^40^ Therefore, it is tempting to speculate that this sex dichotomy in insulin sensitivity might explain why we find a reduction of fetal growth by the intervention in males only. We suggest that future clinical studies should consider neonatal sex as a major determinant of pregnancy outcomes and report outcomes in a sex‐dependent manner.

### 4.1 Strength, limitations

Strengths of our study are its randomised and longitudinal design, encompassing repeated ultrasound measurements from early pregnancy until birth and the multicentre, pan‐European design.

We were not able to adjust analyses for baseline values of fetal growth, as no baseline ultrasound was performed at a fixed gestational age. As most fetal growth parameters were slightly smaller in the UC group in early pregnancy, this might have contributed to an underestimation of intervention effects in later pregnancy.

Clinical indications for ultrasound scans could have also influenced results, as growth of fetuses with a larger number of scans is more precisely estimated. However, the number of ultrasound scans per participant was evenly distributed between randomisation groups and adjustment for these did not change results. We included all ultrasound scan measurements that were available and not just the research standard scans taken as part of the RCT. This is consistent with previous cohort studies of fetal growth^14^, ^15^ and allows more refined trajectory analyses, that is allowing for different rates of growth at different time points. It is possible that measurements from different scanners and with different ultrasound scan systems could introduce some additional variation and imprecision of the results. Another limitation is that we were not able to analyse measurements of fetal fat mass or body composition, as in the LIMIT trial.^25^

As the DALI RCT did not include any lean pregnant women and largely consisted of white European women, results may not be generalisable to women from other ethnic groups and those with a BMI < 29 kg/m^2^.

## 5 CONCLUSION

A pregnancy lifestyle intervention combining PA and HE counselling may attenuate fetal growth in women with obesity with some evidence that this effect might be stronger in male fetuses, although this needs replication. Fetal overgrowth and offspring adiposity are complications of maternal obesity during pregnancy,^12^, ^42^, ^43^ which makes our findings in this obese population relevant for clinical practice, as fetal size was attenuated without increasing the number of SGA babies. These findings underline the importance of promoting a healthy lifestyle during pregnancy. Future research is needed to elucidate underlying pathways and to investigate whether changes in offspring size affect health outcomes on the long term. Special attention should be paid to changes in body composition and adiposity in offspring follow‐up. Because adipose tissue is metabolically active, adiposity is a more relevant parameter for future health than weight or BMI.

## CONFLICT OF INTEREST STATEMENT

No conflict of interest was declared.

## Supporting information


**Data S1.** Supporting information.

## APPENDIX A DALI Core Investigator Group

Gernot Desoye^1^; David Simmons^3^; Rosa Corcoy^11,12^; Juan M. Adelantado Perez^11,12^; Alexandra Kautzky‐Willer^4,13^; Jürgen Harreiter^4,5^; Elizabeth Mathiesen^14,15^; Dorte M. Jensen^16,17^; Lise Lotte T. Andersen^16,17^; Fidelma Dunne^18^; Annunziata Lapolla^15^; Maria G. Dalfra^19^; Alessandra Bertolotto^20^; Mireille N. M. van Poppel^2^; Judith G. M. Jelsma^21,22^; Sander Galjaard^23^; Ewa Wender‐Ozegowska^24^; Agnieszka Zawiejska^25^; David Hill^26^; Roland Devlieger^27^; Frank J. Snoek^22,28^

^11^Institut de Recerca de l'Hospital de la Santa Creu i Sant Pau, Barcelona, Spain

^12^CIBER‐BBN, ISCIII, Madrid, Spain

^13^Gender Institute lapura, Gars am Kamp, Austria

^14^Department of Clinical Medicine, University of Copenhagen, Copenhagen, Denmark

^15^Department of Endocrinology and Center for Pregnant Women with Diabetes, Rigshospitalet, Copenhagen, Denmark

^16^Department of Endocrinology and Department of Gynaecology and Obstetrics, Odense University Hospital, Odense, Denmark

^17^Department of Clinical Research, Faculty of Health Sciences, University of Southern Denmark, Odense, Denmark

^18^Clinical Research Facility and College of Medicine Nursing and Health Sciences, University of Galway, Galway, Ireland

^19^Università Degli Studi di Padova, Padua, Italy

^20^Azienda Ospedaliero Universitaria Pisa, Pisa, Italy

^21^Department of Public and Occupational Health, Amsterdam UMC, Vrije Universiteit Amsterdam, Amsterdam, Netherlands

^22^Amsterdam Public Health Research Institute, Health Behaviours & Chronic Diseases, Amsterdam, Netherlands

^23^Department of Obstetrics and Gynaecology, Division of Obstetrics and Prenatal Medicine, Erasmus MC, University Medical Centre Rotterdam, Rotterdam, Netherlands

^24^WO‐Department of Reproduction, Chair of Feto‐maternal Medicine, Poznan University of Medical Sciences, Poznan, Poland

^25^Medical Faculty I, Poznan University of Medical Sciences, Poznan, Poland

^26^Lawson Health Research Institute, London, Ontario, Canada

^27^KU Leuven Department of Development and Regeneration: Pregnancy, Fetus and Neonate, Gynaecology and Obstetrics, University Hospitals Leuven, Leuven, Belgium

^28^Department of Medical Psychology, Amsterdam UMC, Vrije Universiteit Amsterdam, Amsterdam, Netherlands
