# Supplementary material for: Effect of a physical activity and healthy eating lifestyle intervention in pregnancy on fetal growth trajectories: The DALI randomised controlled trial
Source: Pediatr Obes. 2025 Jan 19;20(5):e13199. doi: 10.1111/ijpo.13199 (PMC12001303; doi:10.1111/ijpo.13199)
Supplement: Supplementary file 1 — Data S1. Supporting information. [file IJPO-20-e13199-s001.docx]

**Effect of a physical activity and healthy eating lifestyle intervention in pregnancy on fetal growth trajectories: the DALI randomised controlled trial**

Anna M. Dieberger, MSc^1^; Mireille N.M. van Poppel, PhD^2^; Gernot Desoye, PhD^1^; David Simmons, MD (Cantab)^3^; Jürgen Harreiter, MD, PhD^4,5^; Roland Devlieger, MD, PhD^6,7^; Carmen Medina, PhD^8^; Deborah A. Lawlor, PhD^9,10^; Ahmed Elhakeem, PhD^9,10^; on behalf of the DALI core investigator group

Corresponding author:
Mireille van Poppel

Department of Human Movement Science, Sport and Health,

University of Graz, Graz, Austria

E-Mail: mireille.van-poppel@uni-graz.at [Phone: +43 316 380 2335](mailto:)

**Table of Content:**

**Table S1:** Maternal and neonatal characteristics of included and excluded participants

**Table S2**: Number of available observations per period of gestation for analysis of estimated fetal weight (EFW)

**Table S3:** Maternal and neonatal characteristics of participants with different numbers of fetal ultrasound measurements

**Table S4:** Predicted mean fetal size across gestation by randomisation group

**Table S5:** Predicted mean difference in fetal size across gestation by randomisation group stratified by fetal sex (males)

**Table S6:** Predicted mean difference in fetal size across gestation by randomisation group stratified by fetal sex (females)

**Table S7:** Predicted mean difference in fetal size comparing usual care to interventions

**Table S8:** Sensitivity analysis for predicted mean difference in fetal size across gestation adjusted for number of available offspring measurements

**Table S9:** Predicted mean difference in fetal size across gestation adjusted for type of measurement

**Figure S1:** Predicted mean fetal growth trajectories across gestation

**Figure S2:** Predicted mean fetal growth trajectories across gestation by randomisation group

**Figure S3:** Intervention effect on fetal size across gestation by fetal sex

**Figure S4:** Combined intervention effect on fetal size across gestation

**Table S1: Maternal and neonatal characteristics of included and excluded participants (No. = 436)**

| **Variables** | **No.** | **Included (No. = 384)** | **No.** | **Excluded (No. = 52)** | **p-value** |
| --- | --- | --- | --- | --- | --- |
| **Maternal characteristics** |  |  |  |  |  |
| Age, mean (SD), years | 384 | 32.0 (5.3) | 52 | 31.9 (5.8) | 0.878 |
| Multiparous, No. (%) | 384 | 194 (51) | 52 | 27 (52) | 0.966 |
| Higher education, No. (%) | 384 | 210 (55) | 52 | 29 (56) | >0.999 |
| European descent, No. (%) | 384 | 328 (85) | 52 | 50 (96) | 0.055 |
| Living with partner, No. (%) | 384 | 363 (95) | 52 | 47 (90) | 0.383 |
| Smoking, No. (%) | 383 | 63 (16) | 52 | 4 (8) | 0.151 |
| Alcohol consumption, No. (%) | 383 | 22 (6) | 52 | 1 (2) | 0.409 |
| Height, mean (SD), cm | 384 | **165.3 (6.8)** | **52** | **168.1 (6.3)** | **0.005** |
| Pre-pregnancy BMI, median (IQR), kg/m^2^ | 384 | 33.0 (31.2, 35.2) | 52 | 33.0 (31.2, 35.2) | 0.495 |
| GWG at 35-37 weeks, mean (SD), kg | 327 | 8.9 (4.2) | 33 | 8.9 (4.2) | 0.149 |
| GDM at 24-28 or 35-37 weeks, No. (%) | 328 | 124 (38) | 34 | 9 (26) | 0.264 |
| Hypertensive disorders of pregnancy, No. (%) | 347 | 48 (14) | 24 | 2 (8) | >0.999 |
| Randomisation group, No. (%) | 384 |  | 52 |  | 0.780 |
| UC |  | 91 (24) |  | 14 (27) |  |
| PA |  | 95 (25) |  | 15 (29) |  |
| HE |  | 102 (27) |  | 11 (21) |  |
| PA+HE |  | 96 (25) |  | 12 (23) |  |
| **Neonatal characteristics** |  |  |  |  |  |
| Female sex, No. (%) | 363 | 181 (49.9) | 34 | 18 (53) | 0.870 |
| Gestational age at birth, median (IQR), weeks | 360 | 39.7 (38.9, 40.9) | 35 | 39.7 (38.5, 40.5) | 0.296 |
| Preterm birth (< 37 gestational weeks), No. (%) | 355 | 19 (5) | 24 | 4 (17) | 0.071 |
| Caesarean section, No. (%) | 360 | 117 (33) | 24 | 10 (42) | 0.484 |
| Birthweight, mean (SD), g | 364 | 3489 (546) | 26 | 3412 (601) | 0.490 |
| Small for gestational age, No. (%) | 353 | 25 (7) | 22 | 3 (14) | 0.474 |
| Large for gestational age, No. (%) | 354 | 47 (13) | 22 | 5 (23) | 0.354 |
| Abdominal circumference at birth, mean (SD), mm | 293 | 333 (28) | 14 | 333 (17) | 0.916 |
| Head circumference at birth, mean (SD), mm | **343** | **348 (17)** | **16** | **339 (17)** | **0.039** |

BMI, body mass index; HE, healthy eating; PA, physical activity; PA+HE, physical activity and healthy eating; UC, usual care.

No., number of participants

**Table S2: Number of available observations per period of gestation for analysis of estimated fetal weight (EFW)**

| **GA** | **UC** | **PA** | **HE** | **PA+HE** | **Total** |
| --- | --- | --- | --- | --- | --- |
| <16 weeks | 13 | 9 | 15 | 14 | 51 |
| 16-19 weeks | 23 | 28 | 31 | 22 | 104 |
| 20-23 weeks | 24 | 25 | 17 | 29 | 95 |
| 24-27 weeks | 70 | 65 | 65 | 60 | 260 |
| 28-31 weeks | 22 | 26 | 34 | 37 | 119 |
| 32-35 weeks | 48 | 53 | 60 | 50 | 211 |
| 36-39 weeks | 72 | 86 | 89 | 84 | 331 |
| >40 weeks | 47 | 37 | 42 | 40 | 166 |
| **Total** | 319 | 329 | 353 | 336 | 1337 |

GA, gestational age; HE, healthy eating intervention; PA, physical activity intervention; PA+HE, physical activity and healthy eating intervention; UC, usual care.

**Table S3: Maternal and neonatal characteristics of participants with different numbers of fetal ultrasound scans (No. = 384)**

| **Variables** | **No.** | **1-3 US (No. = 293)** | **No.** | **4-6 US (No. = 60)** | **No.** | **7-10 US (No. = 31)** | **p-value** |
| --- | --- | --- | --- | --- | --- | --- | --- |
| **Maternal characteristics** |  |  |  |  |  |  |  |
| Age, mean (SD), years | 293 | 31.8 (5.2) | 60 | 32.9 (5.8) | 31 | 31.9 (5.3) | 0.358 |
| Multiparous, No. (%) | 293 | 155 (53) | 60 | 29 (48) | 31 | 10 (32) | 0.086 |
| Higher education, No. (%) | 293 | 170 (58) | 60 | 28 (47) | 31 | 12 (39) | **0.048** |
| European descent, No. (%) | 293 | 268 (91) | 60 | 43 (72) | 31 | 17 (55) | **<0.001** |
| Living with partner, No. (%) | 293 | 279 (95) | 60 | 58 (97) | 31 | 26 (84) | **0.022** |
| Smoking, No. (%) | 292 | 44 (15) | 60 | 15 (25) | 31 | 4 (13) | 0.144 |
| Alcohol consumption, No. (%) | 292 | 19 (7) | 60 | 3 (5) | 31 | 0 (0) | 0.322 |
| Height, mean (SD), cm | 293 | 165.7 (6.5) | 60 | 163.3 (7.2) | 31 | 165.5 (7.9) | **0.043** |
| Pre-pregnancy BMI, median (IQR), kg/m^2^ | 293 | 32.9 (30.4, 36.0) | 60 | 33.1 (31.0, 35.1) | 31 | 32.9 (31.5, 36.3) | 0.602 |
| GWG at 35-37 weeks, mean (SD), kg | 241 | 7.5 (4.5) | 57 | 8.4 (4.5) | 29 | 7.4 (5.9) | 0.397 |
| GDM at 24-28 or 35-37 weeks, No. (%) | 244 | 84 (34) | 56 | 26 (46) | 28 | 14 (50) | 0.094 |
| Hypertensive disorders of pregnancy, No. (%) | 257 | 30 (12) | 59 | 9 (15) | 31 | 9 (29) | **0.029** |
| Randomisation group | 293 |  | 60 |  | 31 |  | 0.769 |
| UC |  | 73 (25) |  | 11 (18) |  | 7 (23) |  |
| PA |  | 70 (24) |  | 15 (25) |  | 10 (32) |  |
| HE |  | 80 (27) |  | 15 (25) |  | 7 (23) |  |
| PA+HE |  | 70 (24) |  | 19 (32) |  | 7 (23) |  |
| **Neonatal characteristics** |  |  |  |  |  |  |  |
| Female sex, No. (%) | 272 | 132 (49) | 60 | 34 (57) | 31 | 15 (48) | 0.514 |
| Gestational age at birth, median (IQR), weeks | 269 | 39.6 (38.6, 40.7) | 59 | 40.0 (39.3, 40.9) | 31 | 39.7 (39.1, 40.6) | 0.166 |
| Preterm birth (< 37 gestational weeks), No. (%) | 265 | 17 (6) | 59 | 2 (3) | 31 | 0 (0) | 0.248 |
| Caesarean section, No. (%) | 270 | 89 (33) | 59 | 20 (33) | 31 | 8 (26) | 0.700 |
| Birthweight, mean (SD), g | 273 | 3494 (557) | 60 | 3449 (494) | 31 | 3522 (551) | 0.793 |
| Small for gestational age, No. (%) | 263 | 16 (6) | 59 | 6 (10) | 31 | 3 (10) | 0.456 |
| Large for gestational age, No. (%) | 264 | 30 (11) | 59 | 8 (14) | 31 | 9 (29) | **0.023** |
| Abdominal circumference at birth, mean (SD), mm | 217 | 333 (28) | 46 | 330 (28) | 30 | 336 (28) | 0.661 |
| Head circumference at birth, mean (SD), mm | 256 | 348 (17) | 57 | 344 (16) | 30 | 351 (16) | 0.156 |

BMI, body mass index; GDM, gestational diabetes mellitus; GWG, gestational weight gain, HE, healthy eating intervention; PA, physical activity intervention; PA+HE, physical activity and healthy eating intervention; UC, usual care; US, Number of fetal ultrasound scans.

No., number of participants

**Table S4: Predicted mean fetal size across gestation by randomisation group**

|  | **Predicted mean (95% CI)** | | | |
| --- | --- | --- | --- | --- |
|  | **EFW, g (No. = 377; n = 1337)** | | | |
| **GA** | **UC** | **PA** | **HE** | **PA+HE** |
| 16 weeks | 137.8 (95.9; 179.7) | 160.5 (116.6; 204.4) | 160.4 (119.2; 201.5) | 158.6 (117.3; 200.0) |
| 20 weeks | 312.1 (280.2; 344.0) | 335.4 (303.5; 367.2) | 323.4 (291.0; 355.8) | 329.1 (297.9; 360.3) |
| 24 weeks | 654.1 (615.5; 692.6) | 670.4 (631.0; 709.8) | 649.8 (611.1; 688.6) | 658.9 (620.9; 696.9) |
| 28 weeks | 1242.7 (1195.3; 1290.2) | 1241.1 (1193.3; 1289.0) | 1216.7 (1170.7; 1262.8) | 1223.3 (1176.9; 1269.6) |
| 32 weeks | 2066.6 (2004.5; 2128.8) | 2038.8 (1977.5; 2100.1) | 2016.0 (1957.1; 2074.9) | 2012.5 (1952.1; 2072.9) |
| 36 weeks | 2920.2 (2845.6; 2994.8) | 2874.1 (2800.4; 2947.7) | 2856.9 (2786.5; 2927.3) | 2835.3 (2762.4; 2908.3) |
| 40 weeks | 3622.9 (3534.9; 3710.9) | 3579.5 (3490.8; 3668.3) | 3570.3 (3486.2; 3654.5) | 3523.1 (3436.3; 3609.9) |
|  | **AC, mm (No. = 372; n = 1289)** | | | |
| **GA** | **UC** | **PA** | **HE** | **PA+HE** |
| 16 weeks | 103.6 (100.4; 106.8) | 106.6 (103.3; 109.9) | 104.0 (100.8; 107.1) | 104.9 (101.8; 108.0) |
| 20 weeks | 148.7 (146.0; 151.4) | 151.0 (148.3; 153.7) | 149.2 (146.5; 151.9) | 149.8 (147.2; 152.4) |
| 24 weeks | 195.2 (192.2; 198.3) | 196.7 (193.6; 199.9) | 195.2 (192.1; 198.3) | 195.6 (192.6; 198.6) |
| 28 weeks | 243.9 (240.5; 247.2) | 244.4 (241.0; 247.8) | 242.3 (239.0; 245.6) | 242.6 (239.4; 245.9) |
| 32 weeks | 290.9 (286.8; 295.0) | 290.4 (286.3; 294.4) | 287.6 (283.7; 291.5) | 287.8 (283.9; 291.8) |
| 36 weeks | 324.8 (320.2; 329.3) | 323.0 (318.6; 327.5) | 322.1 (317.8; 326.5) | 321.7 (317.2; 326.1) |
| 40 weeks | 338.0 (332.6; 343.3) | 334.9 (329.5; 340.2) | 340.1 (334.9; 345.3) | 338.0 (332.7; 343.3) |
|  | **HC, mm (No. = 379; n = 1365)** | | | |
| **GA** | **UC** | **PA** | **HE** | **PA+HE** |
| 16 weeks | 121.8 (119.5; 124.1) | 122.7 (120.4; 125.0) | 121.2 (119.0; 123.5) | 121.8 (119.5; 124.0) |
| 20 weeks | 171.8 (169.6; 174.0) | 173.9 (171.7; 176.1) | 172.0 (169.8; 174.2) | 172.3 (170.1; 174.5) |
| 24 weeks | 218.9 (216.5; 221.4) | 221.4 (218.9; 223.8) | 219.4 (217.0; 221.9) | 219.5 (217.1; 221.9) |
| 28 weeks | 261.8 (259.3; 264.4) | 263.4 (260.8; 265.9) | 262.0 (259.5; 264.5) | 261.8 (259.3; 264.3) |
| 32 weeks | 298.9 (296.0; 301.9) | 298.8 (295.9; 301.7) | 298.2 (295.4; 301.1) | 297.9 (295.0; 300.8) |
| 36 weeks | 328.0 (324.9; 331.2) | 327.9 (324.7; 331.0) | 326.9 (323.9; 330.0) | 326.7 (323.6; 329.8) |
| 40 weeks | 348.4 (344.9; 351.8) | 351.3 (347.8; 354.9) | 347.9 (344.5; 351.2) | 348.3 (344.9; 351.7) |
|  | **FL, mm (No. = 376; n = 1043)** | | | |
| **GA** | **UC** | **PA** | **HE** | **PA+HE** |
| 16 weeks | 18.7 (18.1; 19.3) | 19.7 (19.2; 20.3) | 18.8 (18.2; 19.4) | 19.1 (18.5; 19.6) |
| 20 weeks | 30.8 (30.2; 31.4) | 32.2 (31.6; 32.8) | 31.3 (30.7; 31.9) | 31.5 (30.9; 32.1) |
| 24 weeks | 42.3 (41.7; 42.9) | 43.5 (42.9; 44.1) | 42.8 (42.2; 43.4) | 43.0 (42.4; 43.6) |
| 28 weeks | 52.8 (52.1; 53.5) | 53.2 (52.5; 53.8) | 52.9 (52.2; 53.5) | 53.1 (52.4; 53.7) |
| 32 weeks | 61.8 (60.9; 62.6) | 61.5 (60.7; 62.3) | 61.5 (60.7; 62.3) | 61.6 (60.8; 62.4) |
| 36 weeks | 68.7 (67.9; 69.5) | 69.3 (68.5; 70.1) | 69.1 (68.3; 69.8) | 68.7 (68.0; 69.5) |
| 40 weeks | 74.3 (72.6; 76.0) | 76.9 (75.2; 78.5) | 76.0 (74.5; 77.4) | 75.0 (73.5; 76.6) |

See Figure 2 and Figure S2 for graphical presentation of results. Predicted mean growth trajectories of EFW (estimated fetal weight; g), HC (head circumference; mm), AC (abdominal circumference; mm) and FL (femur length; mm) by randomisation group: UC (usual care), PA (physical activity intervention), HE (healthy eating intervention) and PA+HE (physical activity and healthy eating intervention) across gestation. Growth trajectories were estimated using multilevel natural cubic spline models with 2 knots containing an interaction term between gestational age at measurement (continuous; weeks) and randomisation group (categorical; UC, PA, HE, PA+HE). Study site (categorical; Austria, Belgium, Denmark [Copenhagen, Odense], Ireland, Italy [Pisa, Padua], Netherlands, Poland, Spain, United Kingdom) was added to all models as covariate with Spain set as reference category.

No., number of participants; n, number of observations.

**Table S5: Predicted mean difference in fetal size across gestation by randomisation group stratified by fetal sex (males)**

|  | **Predicted mean difference (95% CI) male offspring** | | | |
| --- | --- | --- | --- | --- |
|  | **EFW, g (Total No. = 363; total n = 1320 / No. male = 182; n male = 638)** | | | |
| *GA* | *UC* | *PA* | *HE* | *PA+HE* |
| 16 weeks | REF | -12.7 (-98.3; 72.9) | 25.4 (-61.3; 112.0) | 4.1 (-84.2; 92.4) |
| 20 weeks | REF | 21.9 (-38.0; 81.8) | 18.8 (-38.7; 76.2) | 14.5 (-47.4; 76.5) |
| 24 weeks | REF | 31.3 (-42.3; 105.0) | 3.6 (-66.9; 74.1) | 10.6 (-64.0; 85.2) |
| 28 weeks | REF | 3.6 (-87.4; 94.7) | -24.3 (-111.5; 62.9) | -14.5 (-106.4; 77.3) |
| 32 weeks | REF | -53.9 (-174.2; 66.5) | -62.0 (-177.3; 53.2) | -62.4 (-185.1; 60.3) |
| 36 weeks | REF | -90.2 (-237.0; 56.7) | -90.9 (-232.0; 50.1) | -122.9 (-272.7; 26.9) |
| 40 weeks | REF | -63.1 (-238.4; 112.3) | -95.7 (-263.1; 71.7) | -185.8 (-362.5; -9.2) |
|  | **AC, mm (Total No. = 358; total n = 1272 / No. male = 179; n male = 618)** | | | |
| *GA* | *UC* | *PA* | *HE* | *PA+HE* |
| 16 weeks | REF | 4.3 (-2.0; 10.7) | 1.7 (-4.8; 8.1) | 5.6 (-1.0; 12.2) |
| 20 weeks | REF | 3.5 (-1.5; 8.5) | 1.3 (-3.5; 6.1) | 3.5 (-1.7; 8.6) |
| 24 weeks | REF | 2.4 (-3.4; 8.3) | 0.3 (-5.3; 5.9) | 1.2 (-4.7; 7.1) |
| 28 weeks | REF | 1.1 (-5.2; 7.5) | -1.5 (-7.6; 4.6) | -1.2 (-7.6; 5.2) |
| 32 weeks | REF | -0.5 (-8.2; 7.3) | -3.4 (-10.9; 4.1) | -3.6 (-11.5; 4.3) |
| 36 weeks | REF | -2.1 (-10.9; 6.7) | -3.0 (-11.5; 5.5) | -5.0 (-14.0; 4.0) |
| 40 weeks | REF | -3.7 (-14.2; 6.8) | 1.4 (-8.7; 11.5) | -4.9 (-15.6; 5.8) |
|  | **HC, mm (Total No. = 363; total n = 1346 / No. male = 182; n male = 660)** | | | |
| *GA* | *UC* | *PA* | *HE* | *PA+HE* |
| 16 weeks | REF | 0.5 (-3.5; 4.6) | 0.5 (-3.6; 4.6) | 0.9 (-3.4; 5.1) |
| 20 weeks | REF | 1.8 (-2.1; 5.6) | -0.1 (-3.8; 3.6) | -0.7 (-4.7; 3.3) |
| 24 weeks | REF | 2.1 (-2.2; 6.5) | -0.9 (-5.0; 3.3) | -1.8 (-6.2; 2.7) |
| 28 weeks | REF | 1.2 (-3.3; 5.7) | -1.8 (-6.1; 2.5) | -2.2 (-6.7; 2.4) |
| 32 weeks | REF | -0.5 (-5.8; 4.8) | -2.7 (-7.8; 2.4) | -2.1 (-7.5; 3.3) |
| 36 weeks | REF | -0.6 (-6.3; 5.2) | -3.2 (-8.8; 2.4) | -2.2 (-8.1; 3.7) |
| 40 weeks | REF | 2.8 (-3.5; 9.1) | -3.0 (-9.1; 3.1) | -3.1 (-9.5; 3.2) |
|  | **FL, mm (Total No. = 358; total n = 1019 / No. male = 179; n male = 497)** | | | |
| *GA* | *UC* | *PA* | *HE* | *PA+HE* |
| 16 weeks | REF | 1.1 (0.1; 2.2) | 0.0 (-1.1; 1.0) | 0.3 (-0.8; 1.4) |
| 20 weeks | REF | 1.0 (-0.1; 2.1) | 0.3 (-0.8; 1.3) | 0.1 (-1.1; 1.2) |
| 24 weeks | REF | 0.7 (-0.4; 1.9) | 0.3 (-0.8; 1.4) | -0.1 (-1.3; 1.1) |
| 28 weeks | REF | 0.2 (-1.0; 1.4) | -0.1 (-1.2; 1.1) | -0.1 (-1.3; 1.1) |
| 32 weeks | REF | -0.1 (-1.6; 1.4) | -0.2 (-1.6; 1.2) | 0.1 (-1.4; 1.7) |
| 36 weeks | REF | 0.6 (-0.9; 2.0) | 0.7 (-0.7; 2.1) | 0.5 (-1.0; 2.1) |
| 40 weeks | REF | 2.0 (-1.5; 5.4) | 2.6 (-0.4; 5.5) | 1.1 (-2.4; 4.6) |

See Figure S3 for graphical presentation of results. Predicted mean differences in EFW (estimated fetal weight; g), HC (head circumference; mm), AC (abdominal circumference; mm) and FL (femur length; mm) comparing the interventions PA (physical activity), HE (healthy eating) and PA+HE (physical activity and healthy eating) to UC (usual care; reference group) across gestation, stratified by fetal sex. Mean differences were estimated using multilevel natural cubic spline models with 2 knots containing interaction terms between gestational age at measurement (continuous; weeks), randomisation group (categorical; UC, PA, HE, PA+HE) and fetal sex (male vs. female). Predicted mean differences for males and females are based on the same model, but were estimated separately for each sex. Study site (categorical; Austria, Belgium, Denmark [Copenhagen, Odense], Ireland, Italy [Pisa, Padua], Netherlands, Poland, Spain, United Kingdom) was added to all models as covariate.

No., number of participants; n, number of observations; REF, reference category.

**Table S6: Predicted mean difference in fetal size across gestation by randomisation group stratified by fetal sex (females)**

|  | **predicted mean difference (95% CI) female offspring** | | | |
| --- | --- | --- | --- | --- |
|  | **EFW, g (No. = 363; n = 1320 / No. female = 181; n female = 682)** | | | |
| *GA* | *UC* | *PA* | *HE* | *PA+HE* |
| 16 weeks | REF | 56.1 (-23.7; 136.0) | 29.1 (-44.7; 103.0) | 35.1 (-38.0; 108.2) |
| 20 weeks | REF | 26.2 (-30.2; 82.7) | 3.8 (-55.5; 63.2) | 20.8 (-33.0; 74.6) |
| 24 weeks | REF | 2.7 (-66.4; 71.8) | -19.9 (-91.4; 51.6) | 1.4 (-65.2; 68.1) |
| 28 weeks | REF | -11.5 (-97.7; 74.8) | -41.3 (-128.2; 45.5) | -25.4 (-109.5; 58.7) |
| 32 weeks | REF | -17.0 (-132.8; 98.7) | -57.3 (-172.7; 58.1) | -53.9 (-167.0; 59.2) |
| 36 weeks | REF | -23.7 (-164.7; 117.3) | -59.5 (-199.4; 80.5) | -59.6 (-197.7; 78.5) |
| 40 weeks | REF | -39.8 (-210.7; 131.1) | -42.0 (-211.9; 128.0) | -23.4 (-190.4; 143.5) |
|  | **AC, mm (No. = 358; n = 1272 / No. female = 179; n female = 654)** | | | |
| *GA* | *UC* | *PA* | *HE* | *PA+HE* |
| 16 weeks | REF | 2.4 (-3.5; 8.2) | -0.8 (-6.3; 4.6) | -1.3 (-6.7; 4.0) |
| 20 weeks | REF | 1.5 (-3.3; 6.2) | -0.9 (-5.8; 4.1) | -0.4 (-4.9; 4.2) |
| 24 weeks | REF | 0.7 (-4.8; 6.1) | -1.3 (-7; 4.4) | -0.2 (-5.5; 5.1) |
| 28 weeks | REF | 0.0 (-6.0; 6.0) | -2.3 (-8.4; 3.7) | -1.0 (-6.8; 4.8) |
| 32 weeks | REF | -0.6 (-8.0; 6.9) | -3.3 (-10.8; 4.1) | -2.3 (-9.5; 5.0) |
| 36 weeks | REF | -1.4 (-9.8; 6.9) | -2.4 (-10.7; 6.0) | -1.2 (-9.4; 7.1) |
| 40 weeks | REF | -2.8 (-13; 7.4) | 2.0 (-8.4; 12.4) | 4.2 (-5.7; 14.1) |
|  | **HC, mm (No. = 363; n = 1346 / No. female = 181; n female = 686)** | | | |
| *GA* | *UC* | *PA* | *HE* | *PA+HE* |
| 16 weeks | REF | 1.3 (-2.7; 5.4) | -1.4 (-5.2; 2.3) | -0.5 (-4.2; 3.2) |
| 20 weeks | REF | 2.4 (-1.3; 6.1) | -0.3 (-4.1; 3.5) | 1.8 (-1.8; 5.3) |
| 24 weeks | REF | 2.6 (-1.5; 6.7) | 0.4 (-3.9; 4.7) | 2.7 (-1.3; 6.7) |
| 28 weeks | REF | 1.4 (-2.8; 5.7) | 0.5 (-3.9; 4.8) | 1.7 (-2.4; 5.8) |
| 32 weeks | REF | -0.5 (-5.6; 4.6) | 0.0 (-5.1; 5.1) | -0.5 (-5.4; 4.4) |
| 36 weeks | REF | -0.9 (-6.3; 4.6) | -0.1 (-5.6; 5.3) | -0.9 (-6.3; 4.4) |
| 40 weeks | REF | 2.1 (-4.3; 8.5) | 0.6 (-5.7; 6.9) | 2.7 (-3.4; 8.8) |
|  | **FL, mm (No. = 358; n = 1019 / No. female = 179; n female = 522)** | | | |
| *GA* | *UC* | *PA* | *HE* | *PA+HE* |
| 16 weeks | REF | 1.1 (0.0; 2.1) | 0.1 (-0.9; 1.2) | 0.4 (-0.5; 1.4) |
| 20 weeks | REF | 1.8 (0.7; 2.8) | 0.5 (-0.6; 1.6) | 1.1 (0.1; 2.1) |
| 24 weeks | REF | 1.7 (0.6; 2.8) | 0.6 (-0.6; 1.7) | 1.2 (0.1; 2.2) |
| 28 weeks | REF | 0.6 (-0.6; 1.8) | 0.2 (-1.1; 1.4) | 0.5 (-0.7; 1.6) |
| 32 weeks | REF | -0.4 (-2.0; 1.2) | -0.3 (-1.9; 1.3) | -0.4 (-1.9; 1.1) |
| 36 weeks | REF | 0.7 (-0.7; 2.1) | -0.1 (-1.5; 1.4) | -0.4 (-1.8; 1.0) |
| 40 weeks | REF | 3.6 (0.1; 7.0) | 0.8 (-2.9; 4.5) | 0.4 (-2.9; 3.6) |

See Figure S3 for graphical presentation of results. Predicted mean differences in EFW (estimated fetal weight; g), HC (head circumference; mm), AC (abdominal circumference; mm) and FL (femur length; mm) comparing the interventions PA (physical activity), HE (healthy eating) and PA+HE (physical activity and healthy eating) to UC (usual care; reference group) across gestation, stratified by fetal sex. Mean differences were estimated using multilevel natural cubic spline models with 2 knots containing interaction terms between gestational age at measurement (continuous; weeks), randomisation group (categorical; UC, PA, HE, PA+HE) and fetal sex (male vs. female). Predicted mean differences for males and females are based on the same model, but were estimated separately for each sex. Study site (categorical; Austria, Belgium, Denmark [Copenhagen, Odense], Ireland, Italy [Pisa, Padua], Netherlands, Poland, Spain, United Kingdom) was added to all models as covariate.

No., number of participants; n, number of observations; REF, reference category.

**Table S7: Predicted mean difference in fetal size comparing usual care to interventions**

|  | **Predicted mean difference (95% CI)** | |
| --- | --- | --- |
|  | **EFW, g (No. = 377; n = 1337)** | |
| *GA* | *UC* | *Intervention* |
| 16 weeks | REF | 22.2 (-29.3; 73.6) |
| 20 weeks | REF | 17.2 (-23.1; 57.6) |
| 24 weeks | REF | 5.6 (-42.6; 53.9) |
| 28 weeks | REF | -15.8 (-74.1; 42.5) |
| 32 weeks | REF | -44.4 (-118.8; 30.0) |
| 36 weeks | REF | -65.0 (-153.2; 23.2) |
| 40 weeks | REF | -65.4 (-168.8; 38.1) |
|  | **AC, mm (No. = 372; n = 1289)** | |
| *GA* | *UC* | *Intervention* |
| 16 weeks | REF | 1.5 (-2.5; 5.4) |
| 20 weeks | REF | 1.3 (-2.1; 4.7) |
| 24 weeks | REF | 0.6 (-3.2; 4.5) |
| 28 weeks | REF | -0.7 (-4.9; 3.5) |
| 32 weeks | REF | -2.4 (-7.4; 2.6) |
| 36 weeks | REF | -2.6 (-8.1; 3.0) |
| 40 weeks | REF | -0.3 (-6.7; 6.2) |
|  | **HC, mm (No. = 379; n = 1365)** | |
| *GA* | *UC* | *Intervention* |
| 16 weeks | REF | 0.0 (-2.9; 3) |
| 20 weeks | REF | 0.9 (-2; 3.8) |
| 24 weeks | REF | 1.2 (-1.9; 4.3) |
| 28 weeks | REF | 0.6 (-2.7; 3.8) |
| 32 weeks | REF | -0.6 (-4.3; 3.1) |
| 36 weeks | REF | -0.9 (-4.8; 3.0) |
| 40 weeks | REF | 0.7 (-3.5; 5.0) |
|  | **FL, mm (No. = 376; n = 1043)** | |
| *GA* | *UC* | *Intervention* |
| 16 weeks | REF | 0.5 (-0.2; 1.3) |
| 20 weeks | REF | 0.8 (0.1; 1.6) |
| 24 weeks | REF | 0.8 (0; 1.6) |
| 28 weeks | REF | 0.2 (-0.7; 1.1) |
| 32 weeks | REF | -0.2 (-1.3; 0.8) |
| 36 weeks | REF | 0.3 (-0.7; 1.3) |
| 40 weeks | REF | 1.7 (-0.3; 3.6) |

See Figure S4 for graphical presentation of results. Predicted mean differences in EFW (estimated fetal weight; g), HC (head circumference; mm), AC (abdominal circumference; mm) and FL (femur length; mm) comparing all interventions combined to usual care (reference group) across gestation. Mean differences were estimated using multilevel natural cubic spline models with 2 knots containing an interaction term between gestational age at measurement (continuous; weeks) and randomisation group (usual care vs. intervention). Study site (categorical; Austria, Belgium, Denmark [Copenhagen, Odense], Ireland, Italy [Pisa, Padua], Netherlands, Poland, Spain, United Kingdom) was added to all models as covariate.

No., number of participants; n, number of observations; REF, reference category.

**Table S8: Predicted mean difference in fetal size across gestation adjusted for number of available measurements**

|  | **Predicted mean difference (95% CI)** | | | |
| --- | --- | --- | --- | --- |
|  | **EFW, g (No. = 377; n = 1337)** | | | |
| *GA* | *UC* | *PA* | *HE* | *PA+HE* |
| 16 weeks | REF | 22.7 (-48.3; 93.6) | 22.3 (-47.6; 92.2) | 21.0 (-48.0; 89.9) |
| 20 weeks | REF | 23.3 (-34.5; 81.1) | 11.3 (-46.7; 69.4) | 17.3 (-39.3; 73.8) |
| 24 weeks | REF | 16.3 (-49.3; 82.0) | -4.0 (-68.8; 60.7) | 5.2 (-58.6; 69.0) |
| 28 weeks | REF | -1.6 (-77.6; 74.4) | -25.8 (-100.2; 48.6) | -19.1 (-93.3; 55.1) |
| 32 weeks | REF | -27.9 (-121.9; 66.1) | -50.6 (-142.8; 41.6) | -53.9 (-146.7; 38.9) |
| 36 weeks | REF | -46.2 (-156.4; 64.1) | -63.4 (-171.4; 44.7) | -84.7 (-194.0; 24.7) |
| 40 weeks | REF | -43.4 (-172.8; 85.9) | -52.5 (-178.5; 73.6) | -99.5 (-227.1; 28.1) |
|  | **AC, mm (No. = 372; n = 1289)** | | | |
| *GA* | *UC* | *PA* | *HE* | *PA+HE* |
| 16 weeks | REF | 3.0 (-2.4; 8.4) | 0.4 (-5.0; 5.7) | 1.3 (-4.0; 6.5) |
| 20 weeks | REF | 2.3 (-2.4; 7.0) | 0.5 (-4.3; 5.2) | 1.1 (-3.6; 5.7) |
| 24 weeks | REF | 1.5 (-3.7; 6.7) | -0.1 (-5.2; 5.0) | 0.3 (-4.7; 5.4) |
| 28 weeks | REF | 0.6 (-4.9; 6.1) | -1.6 (-7.0; 3.8) | -1.2 (-6.6; 4.1) |
| 32 weeks | REF | -0.5 (-6.9; 5.8) | -3.3 (-9.6; 3.0) | -3.1 (-9.3; 3.2) |
| 36 weeks | REF | -1.8 (-8.7; 5.2) | -2.7 (-9.5; 4.2) | -3.1 (-10.0; 3.8) |
| 40 weeks | REF | -3.1 (-11.1; 4.9) | 2.2 (-5.7; 10.1) | 0.0 (-8.0; 7.9) |
|  | **HC, mm (No. = 379; n = 1365)** | | | |
| *GA* | *UC* | *PA* | *HE* | *PA+HE* |
| 16 weeks | REF | 0.9 (-30; 4.8) | -0.6 (-4.5; 3.3) | 0.0 (-3.9; 3.9) |
| 20 weeks | REF | 2.1 (-1.7; 5.9) | 0.2 (-3.7; 4.0) | 0.6 (-3.2; 4.3) |
| 24 weeks | REF | 2.4 (-1.7; 6.5) | 0.5 (-3.6; 4.6) | 0.6 (-3.4; 4.7) |
| 28 weeks | REF | 1.5 (-2.7; 5.7) | 0.2 (-4.0; 4.3) | 0.0 (-4.1; 4.2) |
| 32 weeks | REF | -0.1 (-4.8; 4.6) | -0.7 (-5.3; 4.0) | -1.0 (-5.6; 3.6) |
| 36 weeks | REF | -0.2 (-5.1; 4.8) | -1.1 (-6.0; 3.8) | -1.3 (-6.2; 3.6) |
| 40 weeks | REF | 3.0 (-2.4; 8.3) | -0.5 (-5.8; 4.7) | 0.0 (-5.3; 5.3) |
|  | **FL, mm (No. = 376; n = 1043)** | | | |
| *GA* | *UC* | *PA* | *HE* | *PA+HE* |
| 16 weeks | REF | 1.1 (0.1; 2.1) | 0.1 (-0.8; 1.1) | 0.4 (-0.5; 1.4) |
| 20 weeks | REF | 1.4 (0.4; 2.4) | 0.5 (-0.5; 1.5) | 0.7 (-0.3; 1.7) |
| 24 weeks | REF | 1.2 (0.1; 2.2) | 0.5 (-0.5; 1.5) | 0.7 (-0.3; 1.7) |
| 28 weeks | REF | 0.3 (-0.7; 1.4) | 0.1 (-1.0; 1.1) | 0.3 (-0.8; 1.3) |
| 32 weeks | REF | -0.3 (-1.5; 1.0) | -0.3 (-1.5; 1.0) | -0.2 (-1.4; 1.1) |
| 36 weeks | REF | 0.6 (-0.6; 1.8) | 0.3 (-0.9; 1.6) | 0.1 (-1.2; 1.3) |
| 40 weeks | REF | 2.5 (0.1; 5.0) | 1.7 (-0.6; 3.9) | 0.8 (-1.6; 3.1) |

Predicted mean differences in EFW (estimated fetal weight; g), HC (head circumference; mm), AC (abdominal circumference; mm) and FL (femur length; mm) comparing the interventions PA (physical activity), HE (healthy eating) and PA+HE (physical activity and healthy eating) to UC (usual care; reference group) across gestation. Mean differences were estimated using multilevel natural cubic spline models with 2 knots containing an interaction term between gestational age at measurement (continuous; weeks) and randomisation group (categorical; UC, PA, HE, PA+HE). Study site (categorical; Austria, Belgium, Denmark [Copenhagen, Odense], Ireland, Italy [Pisa, Padua], Netherlands, Poland, Spain, United Kingdom) was added to all models as covariate. For this sensitivity analysis, number of available offspring measurements (continuous) was added as additional covariate.

No., number of participants; n, number of observations; REF, reference category.

**Table S9: Predicted mean difference in fetal size across gestation adjusted for type of measurement**

|  | **Predicted mean difference (95% CI)** | | | |
| --- | --- | --- | --- | --- |
|  | **EFW, g (No. = 377; n = 1337)** | | | |
| *GA* | *UC* | *PA* | *HE* | *PA+HE* |
| 16 weeks | REF | 21.2 (-39.4; 81.8) | 21.8 (-36.8; 80.4) | 19.9 (-38.8; 78.7) |
| 20 weeks | REF | 23.5 (-21.4; 68.5) | 11.5 (-33.9; 56.8) | 16.9 (-27.6; 61.4) |
| 24 weeks | REF | 17.3 (-37.7; 72.2) | -3.4 (-57.9; 51.1) | 5.3 (-48.7; 59.2) |
| 28 weeks | REF | -1.5 (-68.9; 65.8) | -24.9 (-90.9; 41.2) | -19 (-85.3; 47.2) |
| 32 weeks | REF | -29.6 (-117.1; 57.9) | -49.4 (-135.1; 36.2) | -54.1 (-140.9; 32.7) |
| 36 weeks | REF | -47.7 (-152.9; 57.5) | -61.9 (-165.1; 41.3) | -85.4 (-190.2; 19.4) |
| 40 weeks | REF | -40.4 (-184.2; 103.5) | -50.2 (-191.1; 90.6) | -100.6 (-242.2; 41.0) |
|  | **AC, mm (No. = 372; n = 1289)** | | | |
| *GA* | *UC* | *PA* | *HE* | *PA+HE* |
| 16 weeks | REF | 2.7 (-1.7; 7.1) | 0.3 (-4.0; 4.6) | 1.1 (-3.2; 5.4) |
| 20 weeks | REF | 2.4 (-1.3; 6.1) | 0.5 (-3.2; 4.3) | 1.1 (-2.6; 4.8) |
| 24 weeks | REF | 1.7 (-2.6; 6.0) | 0.1 (-4.2; 4.3) | 0.5 (-3.7; 4.7) |
| 28 weeks | REF | 0.5 (-4.2; 5.2) | -1.5 (-6.1; 3.2) | -1.1 (-5.7; 3.5) |
| 32 weeks | REF | -1.1 (-6.8; 4.6) | -3.4 (-9.0; 2.3) | -3.1 (-8.7; 2.6) |
| 36 weeks | REF | -2.4 (-8.8; 4.0) | -2.9 (-9.2; 3.4) | -3.5 (-9.9; 2.9) |
| 40 weeks | REF | -2.8 (-12.0; 6.4) | 1.7 (-7.4; 10.7) | -0.9 (-10.0; 8.2) |
|  | **HC, mm (No. = 379; n = 1365)** | | | |
| *GA* | *UC* | *PA* | *HE* | *PA+HE* |
| 16 weeks | REF | 0.9 (-2.3; 4.1) | -0.6 (-3.8; 2.6) | 0.0 (-3.2; 3.2) |
| 20 weeks | REF | 2.0 (-1.1; 5.1) | 0.1 (-3.0; 3.2) | 0.5 (-2.6; 3.6) |
| 24 weeks | REF | 2.4 (-1.0; 5.8) | 0.4 (-2.9; 3.8) | 0.6 (-2.8; 3.9) |
| 28 weeks | REF | 1.6 (-2.0; 5.1) | 0.1 (-3.4; 3.6) | 0.0 (-3.5; 3.5) |
| 32 weeks | REF | 0.1 (-4.0; 4.2) | -0.6 (-4.7; 3.4) | -1.0 (-5.1; 3.1) |
| 36 weeks | REF | 0.0 (-4.5; 4.5) | -1.1 (-5.5; 3.4) | -1.2 (-5.7; 3.2) |
| 40 weeks | REF | 2.7 (-3.4; 8.8) | -0.5 (-6.4; 5.5) | 0.1 (-5.9; 6.0) |
|  | **FL, mm** | | | |
| *GA* | *UC* | *PA* | *HE* | *PA+HE* |
| 16 weeks | - | - | - | - |
| 20 weeks | - | - | - | - |
| 24 weeks | - | - | - | - |
| 28 weeks | - | - | - | - |
| 32 weeks | - | - | - | - |
| 36 weeks | - | - | - | - |
| 40 weeks | - | - | - | - |

Predicted mean differences in EFW (estimated fetal weight; g), HC (head circumference; mm) and AC (abdominal circumference; mm) comparing the interventions PA (physical activity), HE (healthy eating) and PA+HE (physical activity and healthy eating) to UC (usual care; reference group) across gestation. Mean differences were estimated using multilevel natural cubic spline models with 2 knots containing an interaction term between gestational age at measurement (continuous; weeks) and randomisation group (categorical; UC, PA, HE, PA+HE). Study site (categorical; Austria, Belgium, Denmark [Copenhagen, Odense], Ireland, Italy [Pisa, Padua], Netherlands, Poland, Spain, United Kingdom) was added to all models as covariate. For this sensitivity analysis, type of measurement (fetal ultrasound scan vs. measurement at birth) was added as additional covariate. As FL (femur length) was measured only during pregnancy, no sensitivity analysis was performed.

No., number of participants; n, number of observations; REF, reference category.

**Figure S1: Predicted mean fetal growth trajectories across gestation**


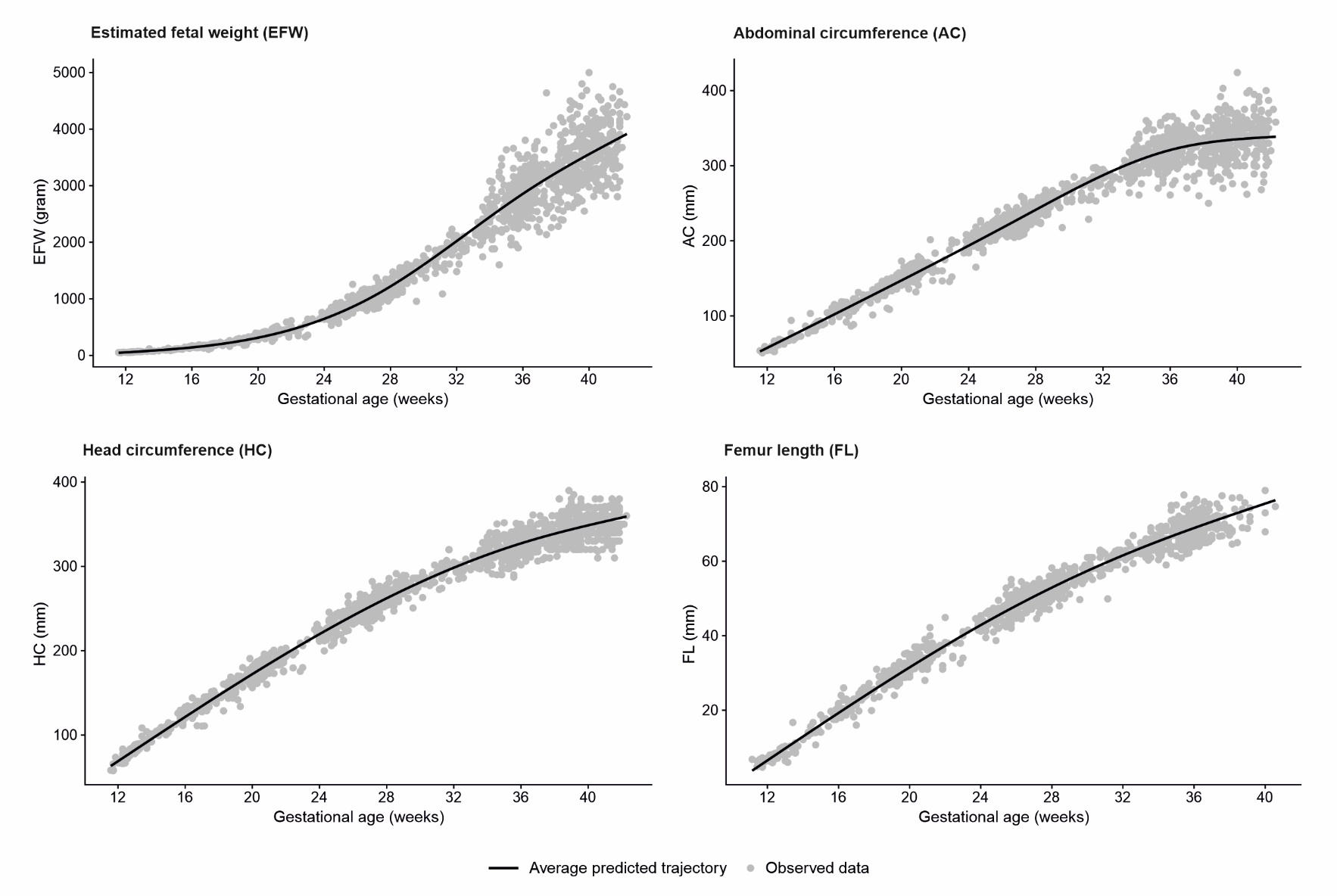


Observed data and predicted mean growth trajectories of EFW (estimated fetal weight; g), HC (head circumference; mm), AC (abdominal circumference; mm) and FL (femur length; mm across gestation. Growth trajectories were estimated using multilevel natural cubic spline models with 2 knots. Study site (categorical; Austria, Belgium, Denmark [Copenhagen, Odense], Ireland, Italy [Pisa, Padua], Netherlands, Poland, Spain, United Kingdom) was added to all models as covariate with Spain set as reference category.

**Figure S2: Predicted mean fetal growth trajectories across gestation by randomisation group**

**
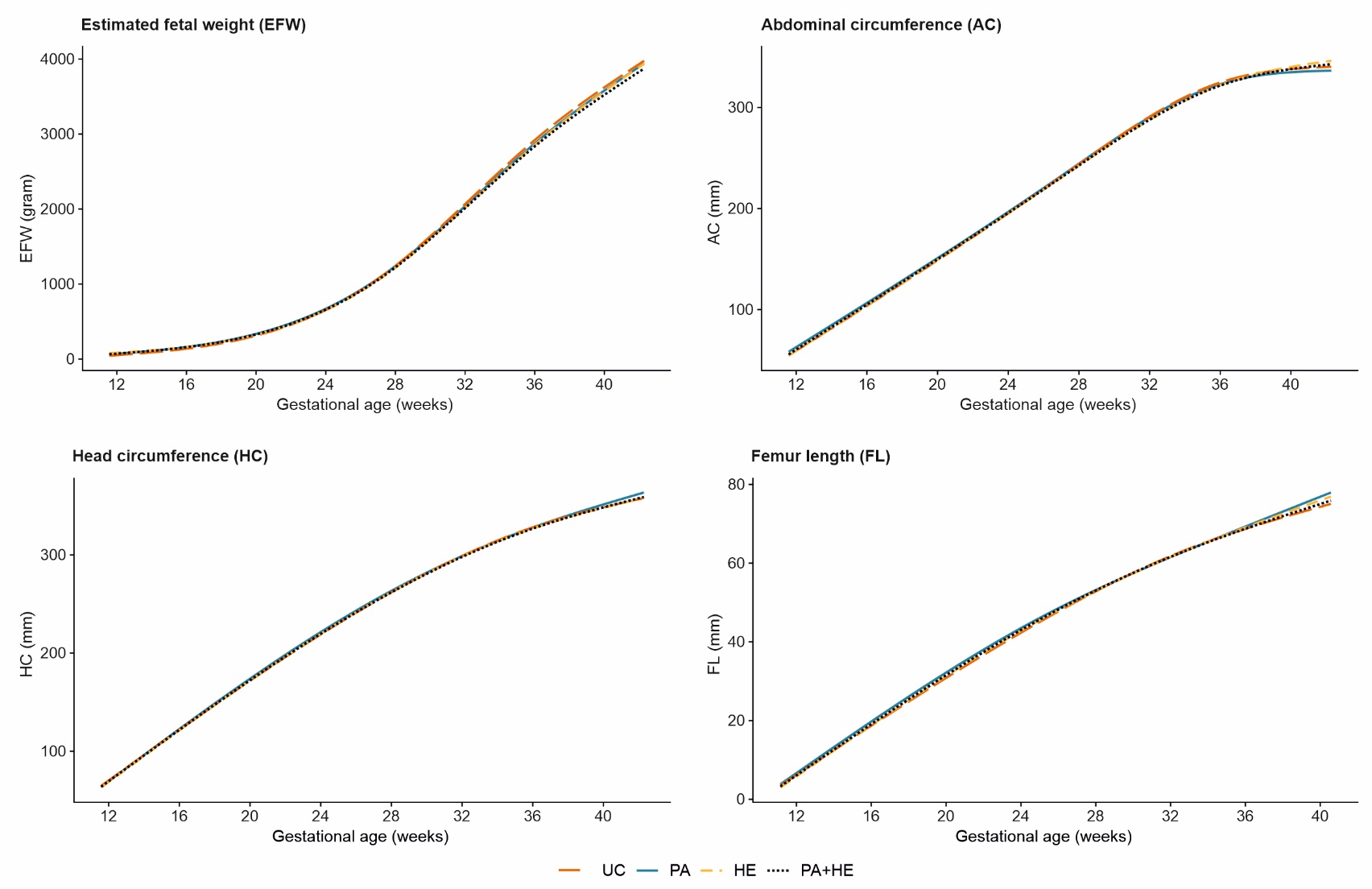
**

Predicted mean growth trajectories of EFW (estimated fetal weight; g), HC (head circumference; mm), AC (abdominal circumference; mm) and FL (femur length; mm) stratified by randomisation group: UC (usual care), PA (physical activity intervention), HE (healthy eating intervention) and PA+HE (physical activity and healthy eating intervention) across gestation. Growth trajectories were estimated using multilevel natural cubic spline models with 2 knots containing an interaction term between gestational age at measurement (continuous; weeks) and randomisation group (categorical; UC, PA, HE, PA+HE). Study site (categorical; Austria, Belgium, Denmark [Copenhagen, Odense], Ireland, Italy [Pisa, Padua], Netherlands, Poland, Spain, United Kingdom) was added to all models as covariate with Spain set as reference category.

**Figure S3: Intervention effect on fetal size across gestation by fetal sex**

**
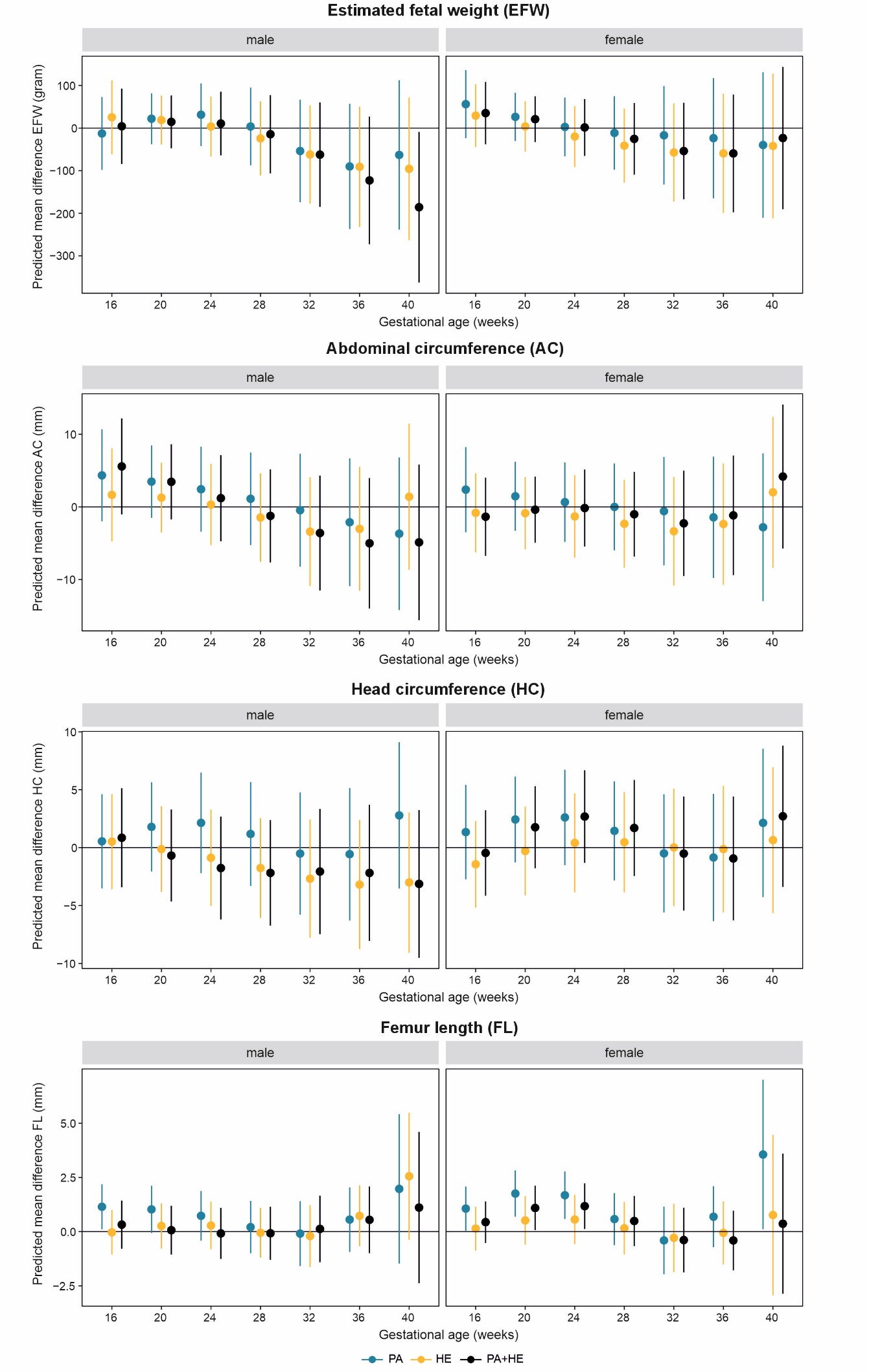
**

Graphical presentation of Table S4 – S5. Predicted mean differences in EFW (estimated fetal weight; g), HC (head circumference; mm), AC (abdominal circumference; mm) and FL (femur length; mm) comparing the interventions PA (physical activity), HE (healthy eating) and PA+HE (physical activity and healthy eating) to UC (usual care; reference group) across gestation, stratified by fetal sex. Mean differences were estimated using multilevel natural cubic spline models with 2 knots containing interaction terms between gestational age at measurement (continuous; weeks), randomisation group (categorical; UC, PA, HE, PA+HE) and fetal sex (male vs. female). Study site (categorical; Austria, Belgium, Denmark [Copenhagen, Odense], Ireland, Italy [Pisa, Padua], Netherlands, Poland, Spain, United Kingdom) was added to all models as covariate.

**Figure S4: Combined intervention effect on fetal size across gestation**


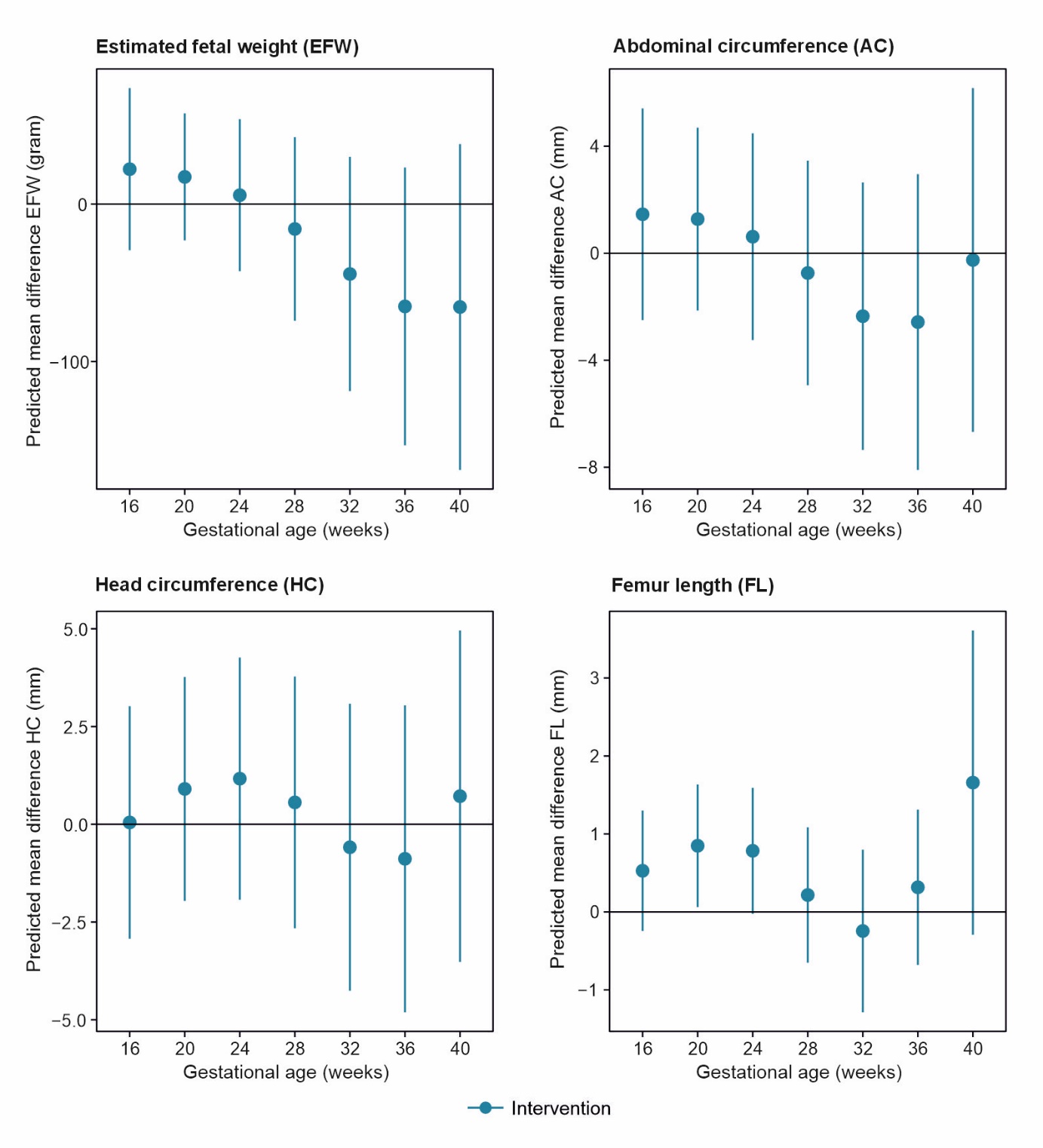


Graphical representation of Table S6. Predicted mean differences in EFW (estimated fetal weight; g), HC (head circumference; mm), AC (abdominal circumference; mm) and FL (femur length; mm) comparing all interventions combined to usual care (reference group) across gestation. Mean differences were estimated using multilevel natural cubic spline models with 2 knots containing an interaction term between gestational age at measurement (continuous; weeks) and randomisation group (usual care vs. intervention). Study site (categorical; Austria, Belgium, Denmark [Copenhagen, Odense], Ireland, Italy [Pisa, Padua], Netherlands, Poland, Spain, United Kingdom) was added to all models as covariate.
